# Automated Method for the Sensitive Analysis of Volatile Amines in Seawater

**DOI:** 10.1021/acsestwater.4c00007

**Published:** 2024-05-03

**Authors:** Preston Chebai Akenga, Mark F. Fitzsimons

**Affiliations:** Biogeochemistry Research Centre, School of Geography, Earth and Environmental Sciences, University of Plymouth, Drake Circus,Plymouth PL4 8AA, U.K.

**Keywords:** methylamines, headspace-solid-phase microextraction, automation, optimization

## Abstract

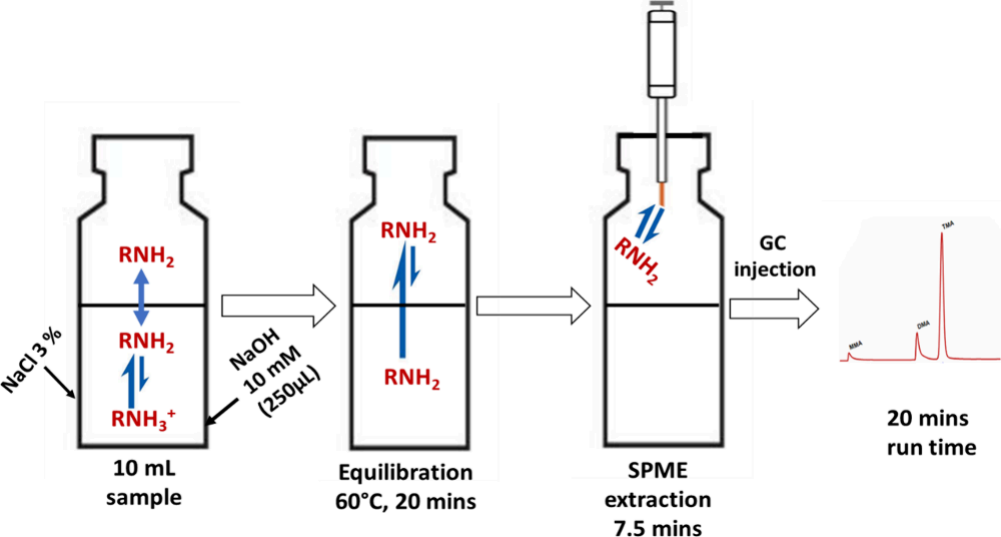

Methylamines are polar, volatile, and organic nitrogen-containing compounds. They are challenging to analyze, limiting our understanding of their occurrence and role within the marine nitrogen cycle. We describe an automated headspace solid-phase microextraction method, coupled with gas chromatography and nitrogen phosphorus detection (HS-SPME-GC-NPD), for analyzing methylamines in seawater. Three SPME conditions were investigated: temperature, equilibration, and extraction. The method was 6–24 times more sensitive to trimethylamine (TMA) than to dimethylamine (DMA) and monomethylamine (MMA). DMA and TMA were detected in small seawater volumes (2.5–10 mL), at volumes 100–400 times that previously reported. Detection limits of 19.1, 6.6, and 4.1 nM (nMol L^–1^) for MMA, DMA, and TMA, respectively, were measured in 10 mL sample volumes. Sample throughput was 4–6 times greater than previously reported similar methods. According to the Blue Applicability Grade Index (BAGI) metric, the method was considered “practical” and scored 62.5. The method was used to measure methylamines in seawater samples collected from the Southern Ocean. DMA and TMA were detected at concentrations from < LoD-35 nM and < LoD-48 nM, respectively. This study offers a systematic and standardized method for MA analysis in seawater and can significantly advance understanding of their role in marine systems.

## Abstract 2

Existing methods for methylamines’ measurement are manual and offline. We report an automated SPME preconcentration and extraction procedure, utilizing a small sample volume to achieve sensitive methylamine analysis.

## Introduction

Methylamines (MAs) are low molecular weight, organic nitrogen compounds ubiquitous in marine environments.^1,2^ Recognized roles for the MAs in the marine nitrogen cycle include their remineralization as a source of nitrogen and carbon for microbes^3^ and a source of base to the atmosphere, which contributes to new particle formation.^4−6^ As marine volatiles, oceanic losses of MAs via the sea–air interface could impact atmospheric chemistry by forming cloud condensation nuclei (CCN).^6^ Despite their abundance and environmental significance, little is known about MA production, distribution, and fate,^7^ while fluxes are also poorly characterized.^8,9^ Interest in the understanding of MA occurrence and cycling has increased in the past decade, and one approach proposed to bridge knowledge gaps is a robust assessment of existing analytical techniques for aqueous analysis, including preconcentration steps.^10^

Various analytical technologies and methodologies have been proposed for the analysis of MAs. These include head space-solid phase microextraction coupled with nitrogen selective gas chromatography (HS-SPME-GC-NPD), microdiffusion-GC-NPD, flow injection-GC-NPD, flow injection gas diffusion-ion chromatography (FIGD-IC), and high-performance liquid chromatography with ultraviolet detection (HPLC-UV). These techniques have been used to analyze MAs in marine environments, including atmospheric, sediment, and aqueous samples.^1,7,11−13^

Analytical challenges in analyzing MAs include their low concentrations (nM) in coastal and ocean water, high solubilities, sorption of protonated MAs, and the high ionic concentration of the saline matrix.^1,2,14,15^ Conventionally, MA extraction and preconcentration have required seawater volumes of 500–1000 mL.^14,16^ These volumes create a challenge in sample collection, storage, and transport, particularly in remote environments. Additionally, reported extraction times for SPME could limit sample size and replication.^7^

This study investigated the SPME step of the HS-SPME-GC-NPD previously reported by Cree et al.,^7^ namely, equilibration (incubation) temperature, extraction time, and extraction temperature. A novel aspect of the study was the integration of automation within the analytical procedure, separating the equilibration and extraction steps for SPME and significantly reducing the sample volume. The Blue Applicability Grade Index (BAGI)^17^ was used to evaluate the practicality of the SPME approach, focusing on 10 main attributes. The method was validated by using seawater samples collected from the Southern Ocean.

## Materials and Methods

### Preparation of Standard Solutions

MMA (99%, CAS 74-89-5), DMA (99%, CAS 124-40-3), and TMA (98%, CAS 75-50-3) were purchased in hydrochloride form ([(CH_3_)_*n*_NH_*n*_^+^ Cl^–^]), along with cyclopropylamine (CPA 99%, CAS 765-30-0), analytical grade HCl (37%), 10 M NaOH solution (CAS 7647-14-5), and analytical grade NaCl (7647-14-5). All chemicals were purchased from Thermo Fisher Scientific, UK. Glass vials (20 mL) and screw caps for SPME were purchased from Thermo Fisher Scientific (part numbers 6ASV20-1 and 6ASC18-CTP, respectively).

Glassware was soaked for 24 h in Decon solution (2%), rinsed with high-purity water (HPW; 18.2 MΩ cm), and then immersed in a bath of HCl (10%) for 24 h. Finally, the apparatus was rinsed with HPW, wrapped in foil, and dried in an oven for 2 h (150 °C).

Stock standard solutions of the MAs were prepared at 7.4, 6.1, and 5.2 mM (MMA, DMA, and TMA, respectively) through the accurate dissolution of their hydrochloride salts in HPW. Stock and working solutions were acidified with concentrated HCl at a ratio of 1:1000 v/v (acid:solution). Calibration solutions of 7.4–74.0, 6.1–61.3, and 5.2–52.3 nM were prepared for MMA, DMA, and TMA, respectively. Calibration solutions and samples were prepared in glass vials with screw caps that were compatible with an RSH Triplus autosampler that was used. Specifically, aliquots (10 mL) of the solutions were pipetted into 20 mL glass vials and saturated with NaCl (33% w/v). CPA was used as an internal standard (IS) and was added to each vial to a final concentration of 8.7 nM. The pH of the solution was adjusted to >13.0 by adding 10 M NaOH solution (250 μL), and the vials were immediately sealed.

Working solutions were prepared in triplicate. Blank samples comprised HPW treated with NaCl and NaOH as described. Stock and working standard solutions were prepared regularly.

### Seawater Collection

Seawater volumes of 0.05–1 L were collected using CTD or underway sampling procedures. Samples were collected and filtered through 0.7 μm glass fiber filters (GF/F). Filtered water was immediately acidified at a ratio of 1:1000 v/v (acid:solution), and a headspace was excluded to maintain MAs in majority cationic form. The preserved samples were stored in a refrigerator at 4 °C prior to analysis. Where needed, samples were transported to Plymouth, England, under chilled conditions.

### SPME Variables and Selection of Sample Volume

Automated online sample extraction and injection were achieved by using a TriPlus RSH autosampler system (Thermo Fisher Scientific). Analytes were extracted onto an SPME fiber after equilibration in an integrated oven followed by injection and exposure of the SPME fiber coated with polydimethylsiloxane/divinylbenzene and dimensions of 65 μm × 10 mm (Merck, UK), in the GC, where the analytes were thermally desorbed in the injector, which contained a base-deactivated liner. The three SPME variables assessed in this study included (i) sample equilibration (incubation) temperature, (ii) equilibration (incubation) time, and (iii) extraction time. The effect of varied sample volumes was also evaluated (Table 1). Conditions held constant during optimization are shown in Table 1. Sample equilibration was achieved by placing the sample vials in a heated solid block under constant agitation. Analyte extraction was achieved by inserting 2 mm of the fiber into the headspace of the vial. The fiber injection depth in the GC injector was 20 mm, and the desorption time in the sample injector port was set to 1 min. The injector temperature was 250 °C, and the fiber pre- and postdesorption times (undertaken before and after sample injection) were 5 min, which overlapped with other SPME functions. Each parameter was assessed through replicate injections (*n* = 5), where each injection was drawn from a separate vial of a mixed amine working solution (52, 62, and 74 nM for MMA, DMA, and TMA, respectively). The solution transferred into the five vials was accurately drawn from a common volumetric flask containing the working solutions. The impact of the sample volume on the sensitivity of the analytical method was evaluated at 2.5, 5.0, and 10.0 mL with a working solution containing 50 nM TMA and seawater samples at similar volumes. The concentrations of NaCl, NaOH, and CPA added to the samples were proportional to the sample volume. The limit of detection (LoD) was calculated based on the calibration curve following ICH 1995 method validation guidelines.^18^ Analysis of variance was determined using IBM SPSS Statistic vs 27.

**Table 1:** Range of SPME Parameters Tested for This Study

			SPME factors held constant			
	SPME condition	optimization range	equilibration temperature (°C)	equilibration time (min)	extraction time (min)	working solution volume (mL)
SPME	equilibration temperature (°C)	40–60 °C		20	5	10
	equilibration time (min)	10–30	60		5	10
	extraction time (min)	2.5–7.5	60	20		10
sample volume	sample volume (mL)	2.5–10.0	60	20	7.5	

### Gas Chromatography

The separation and detection of analytes were performed on a Thermo Scientific Trace 1300 Series gas chromatograph equipped with a RSH TriPlus autosampler (Thermo Fisher Scientific, UK). Analytes were resolved on a 0.32 mm (i.d.) × 60 m CP-Volamine column. Detection was achieved by using a nitrogen–phosphorus detector equipped with a rubidium bead. Detector gases (nitrogen, hydrogen, and zero air) were supplied through Precision Series GC gas generators (Peak Scientific, UK), specifically, a Nitrogen 250-GC N_2_ generator, a Hydrogen 200 H_2_ generator, and a Zero air 1.5 gas generator. Helium (N5.0 grade, BOC, UK) was used as the carrier gas (flow rate of 1.38 mL min^–1^). The flow rates of the detector gases H_2_ and air were 60 and 3.5 mL min^–1^, respectively, while the nitrogen makeup gas had a flow rate of 15 mL min^–1^. The injector and detector temperatures were 250 and 300 °C, respectively. The initial oven temperature was 40 °C, which was held for 2 min. The temperature was then increased to 130 °C at a rate of 10 °C min^–1^ and then to 260 °C at a rate of 50 °C min^–1^, where it was held for 4.4 min. The total run time was 20 min. Data acquisition and processing that yielded peak areas were performed by Thermochromeleon vs 7.3 software.

Seawater samples were prepared the same way as the standard solutions (*n* = 3), but less NaCl (30% w/v) was added to take account of their salinity.

## Results and Discussion

To prevent SPME fiber fouling during analyte extraction, no less than 50% of the vial volume was used as headspace, and only 2 mm of the SPME fiber was exposed during extraction. Lower proportionate headspace volumes, up to 4.5%, were previously reported^7^ to accommodate larger sample volumes.

### Optimized SPME Preconcentration Parameters

**Figure 1:**
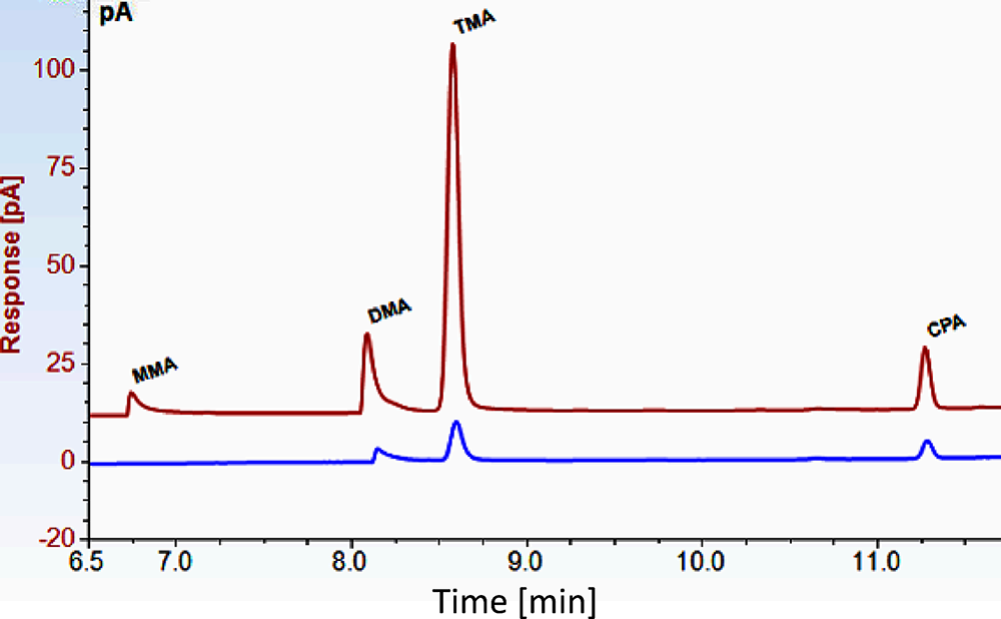
Chromatograms showing (red) standard solution of the MAs (MMA, DMA, and TMA) and internal standard (CPA) at concentrations of 37, 31, 26, and 44 nM, respectively; (blue) chromatogram of a seawater sample.

CPA was selected as an internal standard due to its chemical similarity (volatility and low molecular weight) to the MAs. It elutes at a retention time close to those for the MAs and does not occur naturally in the environment.^19^ NaOH converted most MAs to the gaseous form, shifting the equilibrium to favor their diffusion from solution to headspace for adsorption to the SPME fiber. The three MAs were baseline-resolved on the column and separated from CPA. Retention times for MMA, DMA, TMA, and CPA were 6.7, 8.1, 8.6, and 11.3 min, respectively (Figure 1).

The detector’s sensitivity for the MAs increased from MMA to TMA, consistent with the expected response, where the number of ions produced is expected to be roughly proportional to the number of reduced carbon atoms by the bead.^20^ While it is desirable to obtain perfect Gaussian peaks, in practice, it is rare.^21^ MMA and DMA exhibited peak tailing (prominently in MMA at concentrations <50 nM); however, this was acceptable as the tailing was not accompanied by peak splitting (Figure 1). Previously observed MA peak splitting^7,22^ was attributed to moisture in the headspace during SPME extraction of the analyte.^15^ MAs are highly polar and strongly basic; in their free form, they may decompose in the GC injector port or adsorb to the column, resulting in more than one peak and reduced sensitivity.^12^ The average peak asymmetry for the MMA and DMA chromatographic peaks varied from 2 to 4.5°. TMA and CPA peaks exhibited superior symmetry (1.15–1.40 at all concentrations). There were no interfering peaks bordering MMA and DMA. Consequently, since peak overlap was absent, the detection windows were widened judiciously to improve accuracy in detection and quantification.

**Figure 2:**
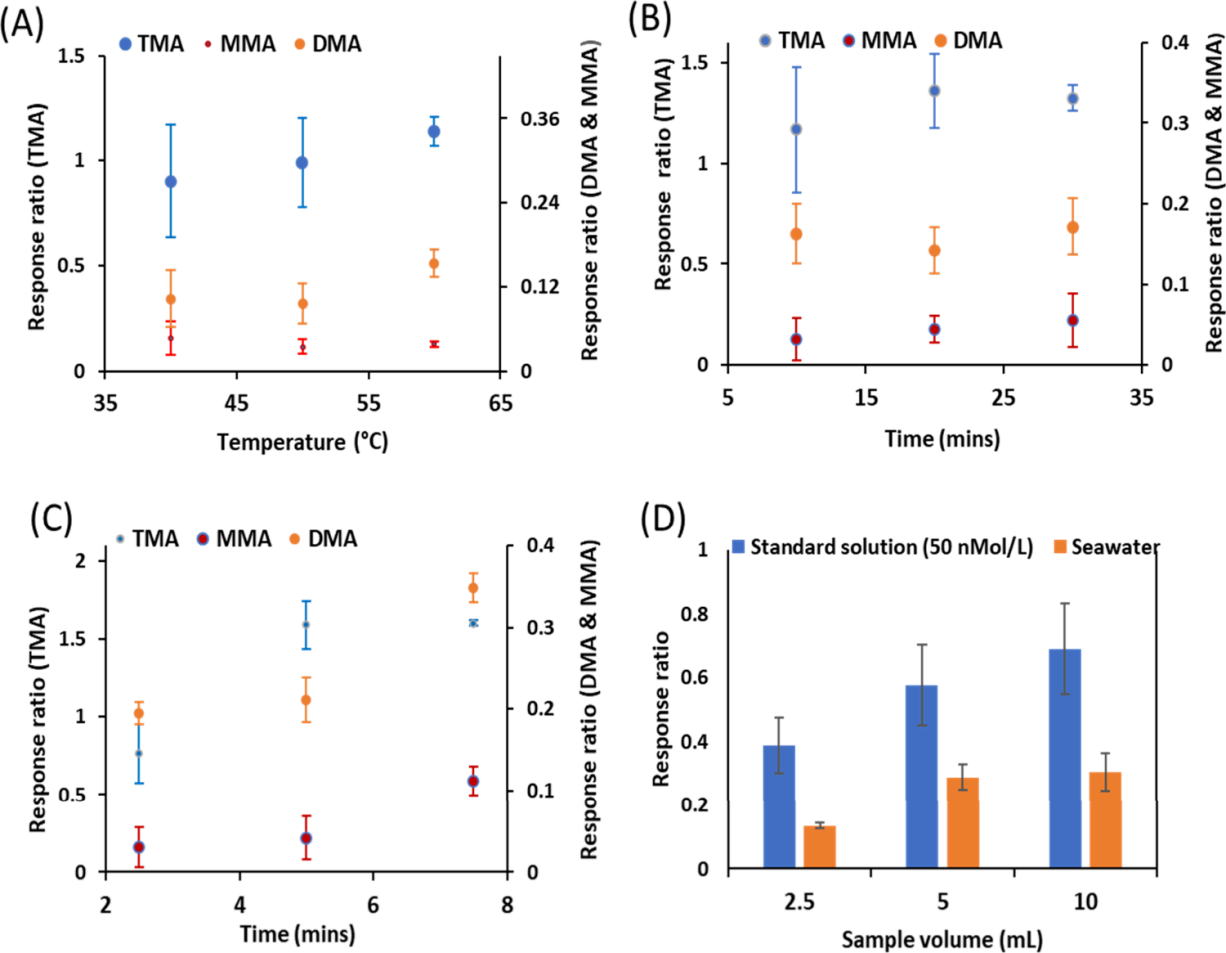
Average response ratios for MMA, DMA, and TMA measured during optimization of (A) equilibration temperature, (B) equilibration time, and (C) extraction time [for panels (A–C), error bars represent % RSDs, *n* = 3] and (D) TMA’s change of response with change in sample volume (2.5–10 mL) in seawater and in high-purity water (50 nM).

The analyte responses to the parameter variations in the SPME process are shown in Figure 2A,C.

Overall, the response for TMA in panels (A–C) was consistently higher (6–24 times) than for DMA and MMA.

### Equilibration Temperature

The data presented in Figure 2A show that analyte response increased with increased equilibration temperature. For example, the response for TMA at 60 °C was 1.25 times higher than that at 40 °C but not significantly different (*p* = 0.407). Similarly, the cumulative mean response for DMA and MMA at 60 °C was 1.2–1.8 times higher than at 40 and 50 °C, respectively. The precision for the three analytes (% RSD) improved as equilibration temperature increased (22.7–6.2, 38.7–12.2, and 29.7–6.1% in MMA, DMA, and TMA, respectively). The variability in response between 50 and 60 min for DMA was significantly different (*p* = 0.02). Based on these data, 60 °C was selected as the optimum equilibration temperature. Equilibration temperature influences the rate of gas diffusion from liquid to headspace.^23^ Temperatures above 60 °C were not tested since high extraction temperatures may lower sample fiber partitioning coefficients, depressing the amount of analyte extracted from the headspace, especially where the analytes in the samples are present at low concentrations.^24^

### Equilibration Time

The data in Figure 2B show that TMA’s precision (% RSD) improved with increased equilibration time, 26.5–4.8%. Unlike TMA, the variability for DMA and MMA was lowest at 20 min. The cumulative mean responses of the three analytes at 20 and 30 min were identical (0.5155 and 0.5173, respectively) and 13% higher than the mean response measured at 10 min (0.4540). The cumulative variabilities of the three analytes measured at 20 and 30 min were 23.9 and 27.1%, respectively, approximately 2 times higher than the precision measured at 10 min (43.7%). For this reason, 20 min was selected as the optimum equilibration time for subsequent analyses. The in-group (intraanalyte) responses for MMA, DMA, and TMA across the three tested times were not significantly different (*p* = 0.493, 0.644, and 0.928, respectively). The measured responses revealed improved equilibration times compared with the 150 min utilized by Cree et al.^7^ The present study’s SPME preconcentration approach contrasted with Cree et al.^7^ in that sample equilibration and extraction steps were sequential.

### Extraction Time

The response across the three evaluated extraction times, namely, 2.5, 5.0, and 7.5 min, is shown in Figure 2C. Individual analyte response increased with extraction time. The cumulative mean responses for the three MAs at 7.5 min were approximately 11 and 51% higher than the responses measured at 5 and 2.5 min, respectively. Method precision similarly improved with increased extraction time, i.e., 80.2–15.7, 7.2–4.9, and 24.6–1.01% for MMA, DMA, and TMA, respectively. For this reason, 7.5 min was selected as the optimum extraction time, mainly due to the measured reproducibility, which is desirable in SPME compared to absolute recoveries, which are secondary.^10^

### Impact of Sample Volume on Analyte Response

Analytically, the selection of sample volume for MA extraction is influenced by the inherent analyte concentrations,^10^ hence the significance of measuring the analyte signal from a range of sample volumes. Generally, analyte response increased with increased sample volume, consistent with the assertion that extraction efficiency is inversely proportional to headspace volume.^24,25^ Common across the two matrices was that the response from the 10 mL samples was 2 times higher and significantly different (*p* = 0.002) than responses from the 2.5 mL samples. The fact that a volume as low as 2.5 mL could yield a measurable analyte signal validated the utilization of a 10 mL sample volume in the present study.

Table 2 contrasts SPME experimental conditions, detection limits, and type and sample sizes between the current study and related studies. Two fibers were used for MA extraction, a PDMS-only fiber for extracting MAs in highly odorous matrix wastewater and PDMS/DVB for seawater samples. The type of fiber is a vital feature in SPME as it significantly impacts the selectivity and sensitivity of a method. PDMS is characteristically nonpolar,^26^ and while the PDMS/DVB fiber is mainly nonpolar, it will extract some polar analytes efficiently^27^ and was the most appropriate fiber for MA extraction despite two of the analytes being outside the reported molecular mass range for the fiber (50–300 Da).^3^

**Table 2:** Reported SPME Methylamine Preconcentration Parameters

					SPME preconcentration parameter					
SPME mode	fiber type	sample/volume	LoD(nM) TMA/DMA/MMA	calibration/linearity	**equilibration temperature (°C)**	**equilibration time (min)**	**extraction time (min)**	total analysis time + (GC run time) (min)	most abundant analyte/other MAs	reference
automated	DVB/PDMS (65 μM)	seawater/10 mL	4.12/6.61/19.1	internal std	60	20	7.5	47.5	TMA	this study
manual	DVB/PDMS (65 μM)	seawater/1 L	0.38–0.89/1.24–2.91/0.48–1.88	external std	60	150^a^		174	TMA	Cree et al.^7^
manual	PDMS (100 μm)	wastewater/20 mL	115	internal std	27	30^a^		66	TMA/ethylamines	Ábalos et al.^12^

a Indicates that equilibration and extraction were concurrent so that separate times could not be recorded.

An *R*^2^ value >0.96 was achieved for the calibration of the three MAs. LoDs of 19.1, 6.7, and 4.1 nM for MMA, DMA, and TMA, respectively, were calculated from a sample volume that was 100 times lower than the 1 L volume reported by Cree et al.^7^ and were comparable (Table 2). The LoDs reported here were 1–2 orders of magnitude lower than a similar extraction and detection method for wastewater^28^ (Table 2). Internal calibration using CPA was utilized in this study to account for the sample matrix and variation in instrument response. The challenges of varying slopes and *x*-intercepts between HPW and seawater-prepared calibration curves have been previously reported.^7^ The method of Cree et al.^7^ (Table 2) had a combined sample extraction and analysis time of 174 min (SPME extraction time of 150 min). In contrast, the total preparation and measurement time achieved in this study were 47.5 min (SPME extraction time of 25 min, equilibration of 20 min, extraction of 7.5 min, and GC analysis of 20 min), which was reduced to 35 min once the RSH autosampler’s overlapping sample preparation function was incorporated. Thus, the present method represents a significant time reduction for sample preparation and analysis time, equivalent to a 4–6 times increase in sample throughput. Meanwhile, in Cree et al.'s work,^7^ nine extractions were achieved in a day, and with automation, a minimum of 40 samples could be analyzed within 24 h.

### Blueness of the SPME Step

The practicality of the SPME step was evaluated using the Blue Applicability Grade Index (BAGI)^17^ metric, whose attributes are listed in Table 3. A number of green metric tools have been proposed for method evaluation, but none considers the practicality of the method, an important parameter that is encountered in routine analysis.^17^

**Table 3:** Ten Parameters Utilized in the Evaluation of the SPME Step Using BAGI Metrics^17^

	BAGI attributes	rating	remarks
1	type of analysis	blue	quantitative, determine amount
2	multi-analyte procedure	light blue	three compounds of the same chemical class
3	analytical technique	light blue	instrument not commonly available in most laboratories
4	number of analytes that are simultaneously determined	light blue	two to six samples can be preconcentrated simultaneously
5	sample preparation	light blue	miniaturized sample preparation involving SPME
6	sample per hour (including pretreatment)	white	47.5 min needed, hence sample per hour needed for a single sample
7	availability of reagents	blue	commercially available reagents, e.g., SPME fiber
8	preconcentration	blue	required sensitivity met with one step, simultaneous sample preparation and preconcentration
9	automation of device	blue	semiautomated with special design systems
10	amount of sample	dark blue	≤10 mL for environmental samples

**Figure 3:**
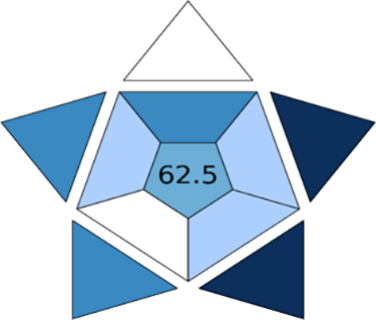
BAGI index pictogram of the SPME extraction step indicating the applicability score.

Table 3 shows the overall assessment of the SPME method using the BAGI metric. For a method to be considered practical, it must obtain a minimum score of 60^17^ and our method had an overall score of 62.5 so it was considered “practical”. In Figure 3, the several shades of blue in the asteroid-shaped pictogram represent varying degrees of compliance: dark blue, blue, light blue, and white represent high compliance, medium compliance, low compliance, and no compliance, respectively. Our method excelled in the sample size and degree of automation. However, compliance was low for the number of samples analyzed (including sample pretreatment) per hour. Feasible and instant improvements could be realized through further reductions in equilibration and extraction time to achieve analysis of at least two samples per hour. Similarly, increasing target analytes to at least six compounds by including ethylamines, for example, would increase the method’s applicability and overall score.

### Measurement of Methylamines in Seawater

The automated method was used to analyze MAs in seawater samples collected from the Southern Ocean. TMA was the most abundant analyte, detected in all 26 analyzed samples, with concentrations varying from < LoD-48 nM. DMA was detected in 20% of samples, varying from < LoD-35 nM. MMA was not detected in any samples (Table 4). The occasional low precision, up to 35% RSD measured during sample analysis, was attributed to the complex seawater matrix. Variations of RSD (33%) during the analysis of MAs in wastewater were considered acceptable due to the sample matrix.^28^ Using a similar analytical approach, TMA was the most abundant MA measured in samples from the Western English Channel.^7^

**Table 4:** Concentration (nM) of MAs in Seawater Measured Using HS-SPME-GC-NPD

location	MMA	DMA	TMA	% RSD	ref
Southern Ocean	ND^b^	< LoD-35	< LoD-48	4–35	this study
Western English Channel	3	6	20	NA^a^	Cree et al.^7^
Southern Ocean	ND	ND	6.9	NA	Dall’Osto^8^

a NA, not available.

b ND, not detected.

Similarly, TMA was the only MA species detected in the Southern Ocean at a maximum concentration of 6.9 nM.^8^ Once released by phytoplankton, quaternary amines are degraded by bacteria primarily to TMA,^9^ which is one possible reason why environmental TMA was detected at significantly higher concentrations than DMA and MMA. It is more basic than DMA and MMA and has been identified as a primary generated organic aerosol^16^

It is not uncommon for MMA not to be detected in seawater. The species was detected with the least abundance in the Western English Channel by Cree et al.^7^ and not by Dall’Osto.^8^

## Conclusions

While offline extraction-based methodologies have facilitated the measurement of MAs in marine samples, the automated and online approach used in this study achieved comparable detection limits using much lower sample volumes. Automating the SPME preconcentration steps significantly improved the performance of HS-SPME-GC-NPD as a technique to measure very low concentrations of MAs, particularly DMA and TMA. The increased sample throughput, which was 4–6 times higher than reported methods using SPME, will contribute to an improved understanding of these analytes’ occurrence, fate, and significance in marine systems. Low LoDs were achieved for all MAs in small sample volumes (≤10 mL), matching those previously obtained from much larger volumes of seawater (0.5–1 L). Future developments should focus on modifying or introducing new tools or materials (e.g., fiber type and SPME arrow) to confidently measure the more weakly detected MMA species in seawater. Also, efforts to automate sample processing should ultimately improve the precision and reporting confidence. Finally, the analysis of MAs in lower sample volumes has improved the SPME extraction method’s sustainability, reducing NaCl consumption from 350 g per sample to 3.5 g, while HCl and NaOH are also added at lower amounts.
